# Decoding bee cleptoparasitism through comparative transcriptomics of *Coelioxoides waltheriae* and its host *Tetrapedia diversipes*

**DOI:** 10.1038/s41598-024-56261-5

**Published:** 2024-05-29

**Authors:** Paulo Cseri Ricardo, Maria Cristina Arias, Natalia de Souza Araujo

**Affiliations:** 1 Departamento de Genética e Biologia Evolutiva – Instituto de Biociências, Universidade de São Paulo, São Paulo, Brazil; 2 Unit of Evolutionary Biology & Ecology – Université Libre de Bruxelles, Brussels, Belgium

**Keywords:** Comparative transcriptomics, Brood parasitism, Cuckoo bee, Solitary bees, Behavioural genetics, Gene expression, Molecular ecology

## Abstract

Cleptoparasitism, also known as brood parasitism, is a widespread strategy among bee species in which the parasite lays eggs into the nests of the host species. Even though this behavior has significant ecological implications for the dynamics of several species, little is known about the molecular pathways associated with cleptoparasitism. To shed some light on this issue, we used gene expression data to perform a comparative analysis between two solitary neotropical bees: *Coelioxoides waltheriae*, an obligate parasite, and their specific host *Tetrapedia diversipes*. We found that ortholog genes involved in signal transduction, sensory perception, learning, and memory formation were differentially expressed between the cleptoparasite and the host. We hypothesize that these genes and their associated molecular pathways are engaged in cleptoparasitism-related processes and, hence, are appealing subjects for further investigation into functional and evolutionary aspects of cleptoparasitism in bees.

## Introduction

Parasitism is an interaction between different species, in which one of them, the parasite, benefits at the expense of another, the host^1,2^. Parasitic species may play an important role in the dynamics of natural populations of host species, for instance, they may affect the susceptibility of their hosts to predation, modify their reproductive patterns, and influence the abundance of endemic and introduced species^3,4^. Cleptoparasitism, alternatively known as brood parasitism, refers to a parasitic behavior where the parasite lays eggs into the nests of the host species. Then during its larval stage, the parasite offspring thrives by consuming the food resources that have been provided by the adult host, ultimately leading to the demise of the host larva or egg, as it is either killed or eaten by the parasite larva. Finally, an adult parasite emerges from the host nest^5^.

Cleptoparasitism is widespread in bees (Hymenoptera: Antophila). It is estimated that approximately 13% of the 20,500 bee species in the world^6^ are obligate cleptoparasites^7^. Currently, it has been inferred that this behavior has arisen independently 18 times in four out of nine bee families: three distinct times in Apidae^8–10^; probably five times in Megachilidae^11–13^; at least once in Colletidae^14^; and possibly nine in Halictidae^5,15^. In spite of these multiple and independent origins, most cleptoparasitic bees show important convergent adaptations such as the reduction or complete loss of pollen-collecting (e.g., pollen-manipulating brushes and scopa) and nest-building structures (e.g., basitibial and pygidial plates for ground-nesting species)^5^. Compared to non-parasitic species, many cleptoparasites also have a heavily sclerotized cuticle in addition to spines, ridges, crests, carinae or lamellae protecting them from the jaws or sting of host females^5,7^. Moreover, convergent anatomical and physiological changes in the reproductive system of some cleptoparasitic species have been described, such as a greater number of mature oocytes in the ovaries or more ovarioles per ovary^16^. These adaptations allow parasitic females to lay several eggs in a short period of time^5,7^.

In addition to their evolutionary relevance, cleptoparasitic species also have an ecological value. Cleptoparasitic bees are considered by far the largest protagonist of solitary bee brood mortality among all natural enemies^17^. In this context, Sheffield et al.^18^ suggest that cleptoparasites, especially generalist ones, perform a stabilizing role in bee communities by attacking dominant and abundant host taxa, which may reduce competition among non-parasitic bee species. These authors also argue that cleptoparasites can serve as indicator taxa for assessing the status of the entire bee community, as their diversity and abundance are closely tied to their host species^18^. However, there is still a deep lack of knowledge regarding the general biology, behavior^7^, and particularly the molecular mechanisms underlying the evolution and maintenance of cleptoparasitism. This knowledge gap might be driven by the rarity to find cleptoparasitic bees in nature^7,19^.

The abundance of cleptoparasitic bees often exhibits a positive correlation with the density of available host nests^18,20^, implying that hosts displaying high levels of gregarious behavior are more susceptible to parasitic attacks. In this context, the species *Coelioxoides waltheriae* Ducke, 1908 and *Tetrapedia diversipes* Klug, 1810 comprise a compelling parasite-host species pair to investigate cleptoparasitism. *T. diversipes*, the host species, has a gregarious behavior, building its nests in naturally pre-existing cavities such as holes in wood^21^, and trap-nests. Indeed, *T. diversipes* easy aggregatory behavior in trap-nests allowed the description of the cleptoparasitic behavior of *C. waltheriae*^21^, the first recorded for the *Coelioxoides* genus. *Coelioxoides* and *Tetrapedia* are endemic to the Neotropical region^22^. Both genera have been formerly grouped within the same tribe, Tetrapediini (Apidae), as they share a number of similar morphological traits^23^. However, recent molecular-based phylogenies have placed *Coelioxoides* within a large cleptoparasitic clade (Nomadinae: Apidae) not sister to *Tetrapedia*^8–10,24,25^. *C. waltheriae* is considered to be the main natural enemy of *T. diversipes*^26^ even though it has been also reported to parasitize nests of other *Tetrapedia* species^27,28^.

Recent studies have successfully employed comparative transcriptomic analysis to unveil molecular features related to complex behaviors. For instance, transcriptomics studies in bees have helped to better understand the molecular pathways related to the honeybee (waggle) dance^29^, *Varroa* sensitive hygiene behaviour^30^, olfactory^31^ and visual^32^ learning, diapause^33,34^, and evolution of task division^35–37^. In this context, herein we use gene expression data to perform a comparative analysis between the correspondent reproductive life stages of *C. waltheriae* and its host, *T. diversipes*. We aimed at identifying diverging gene expression patterns between the solitary pollen-collecting host and the cleptoparasitic bee species. We explored the function of enriched diverging pathways and discussed whether these could be related at some level to their ecological interactions. Instead of assuming any of the differences here observed are causative, we focused on relating our findings to molecular processes for which ecological and/or behavioural relevance has been previously described in the literature to shed some light on the molecular mechanisms potentially related to cleptoparasitic behavior.

## Results

### Transcriptome assembly and annotation

We sampled reproductive females of *C. waltheriae* and *T. diversipes* from the same trap-nest location and at correspondent life stages. In total, we used five sample replicates per species, consisting of a pool of whole-body extractions from individuals (see “Materials and methods”).

After transcriptome assemblies and data treatment (see “Materials and methods”) we obtained a set of 18,208 transcripts for *C. waltheriae* and 11,998 for *T. diversipes*. The main quality parameters associated with each assembly are summarized in Table 1.

**Table 1:** Main quality parameters of *C. waltheriae* and *T. diversipes* assemblies.

Metric	*C. waltheriae*	*T. diversipes*
Total transcripts assembled	87,358	17,866^a^
		40,796^b^
Number of clustered SuperTranscripts^c^	18,208	11,998
SuperTranscripts ≥ 1000 nt	10,195	8051
p fragments mapped^d^	0.93176	0.86625
p bases uncovered^e^	0.0328	0.05047
Transrate optimal score^f^	0.5904	0.50711
Complete BUSCOs (%)	93.4	88.5
Missing BUSCOs (%)	4.1	8.2

^a^Number of transcripts obtained after the reference-based assembly.

^b^Number of transcripts obtained after the reference guided de novo assembly.

^c^After removing contaminating sequences.

^d^Proportion of reads pairs that mapped successfully.

^e^Proportion of bases that were not covered by any read.

^f^This discrete score represents the accuracy and completeness of the assembly based on the assessment of alignments between contigs and reads. The minimum score is 0, while the maximum is 1.0. For a description of the components used in the calculation of this metric, see Smith-Unna et al.^38^.

A total of 10,042 (51.2%) and 7447 (61.9%) transcripts in *C. waltheriae* and *T. diversipes,* respectively, blasted (*e*-value < 1e−5) against protein-coding genes in the UniRef90 database. Moreover, 6253 (31.8%) and 2919 (24.2%) transcripts were estimated to be potential non-coding sequences in *C. waltheriae* and *T. diversipes*. It is worth mentioning that 1397 (7.1%) transcripts were identified as potential contaminants in *C. waltheriae* (Supplementary Material 1). Most were attributed to fungi of the family Tubulinosematidae (Microsporidia): *Tubulinosema ratisbonensis* (1113 transcripts), *Anncaliia algerae* (15 transcripts), and Tubulinosematidae itself (10 transcripts). In addition, 95 (0.48%) of *C. waltheriae* transcripts were identified as very similar to plant protein sequences. Overall, the Fabaceae family was the most representative plants among contaminants (52 transcripts), followed by Asteraceae (11 transcripts) and Solanaceae (6 transcripts). Contaminant transcripts of *T. diversipes* have already been described previously^39^. Herein, we identified only 31 (0.25%) transcripts as potential contaminants (Supplementary Material 1), far less than that observed in *C. waltheriae*, which was expected, since the assembly approaches for *T. diversipes* used a reference genome.

### Differential orthologs expression

**Figure 1:**
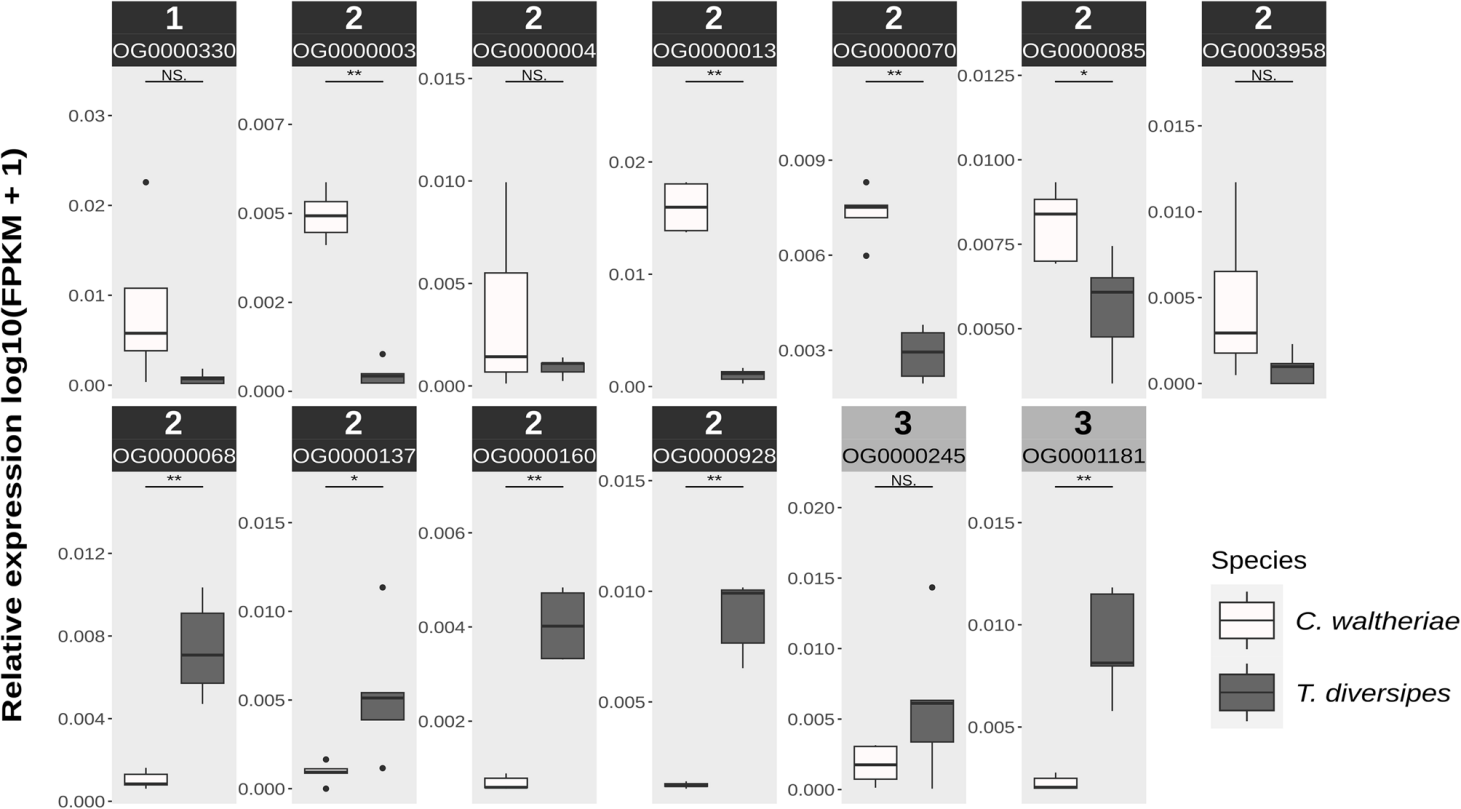
Relative expression of orthologs differentially expressed between the cleptoparasite *Coelioxoides waltheriae* and their host *Tetrapedia diversipes* that were identified as homologs to PiggyBac Transposable Element-derived (1: PGBD2-like; 2: PGBD4-like) and Major Royal Jelly Protein (3) sequences. The respective orthogroups (OGs) are identified according to their IDs in Supplementary Material 3.

Using Orthofinder^40,41^ we identified 4859 orthogroups shared between *C. waltheriae* and *T. diversipes*, of which 3011 were reported as single-copy orthologues. Using blastp^42,43^ searches to identify sequences with the highest homology within common orthogroups we additionally selected 1848 one-to-one orthologs, totaling 4154 one-to-one ortholog pairs between the two species. Of these, around half (2096 or 50.45%) were annotated with at least one functional GO term. After correcting for sampling batch effects accounting for species, the number of samples and sampling year (Supplementary Material 2), the significantly differentially expressed orthologs between the species were obtained by overlapping the results from two strategies (1. TMM normalized counts^44^ and edgeR 3.34.0^45^; 2. Scale Based Normalization [SCBN] method^46^ and NOISeq^47,48^). We identified a total of 646 orthologs as differentially expressed (DE) between the two species (Supplementary Material 3), 335 of which were highly expressed in *C. waltheriae,* and 311 were highly expressed in *T. diversipes.* Among the annotated DE orthologs, two groups caught our attention prior to GO Enrichment Analysis due to their frequency and function. First, a group of eleven transcripts homologous to sequences of PiggyBac transposable element-derived protein genes (*Pgbd4* = 10 transcripts and *Pgbd2* = 1 transcript), most of which (7) were highly expressed in *C. waltheriae* (Fig. 1). Moreover, considering the entire set of transcripts assembled, we found that *C. waltheriae* has more transcripts (n = 68; 0.34% of the transcriptome) of *Pgdb*-like genes than *T. diversipes* (n = 24; 0.19%). Additionally, we found in *T. diversipes* two sequences identified as homologous to Major Royal Jelly Protein/yellow-like (*MRJP/y*) that were highly expressed (Fig. 1).

**Figure 2:**
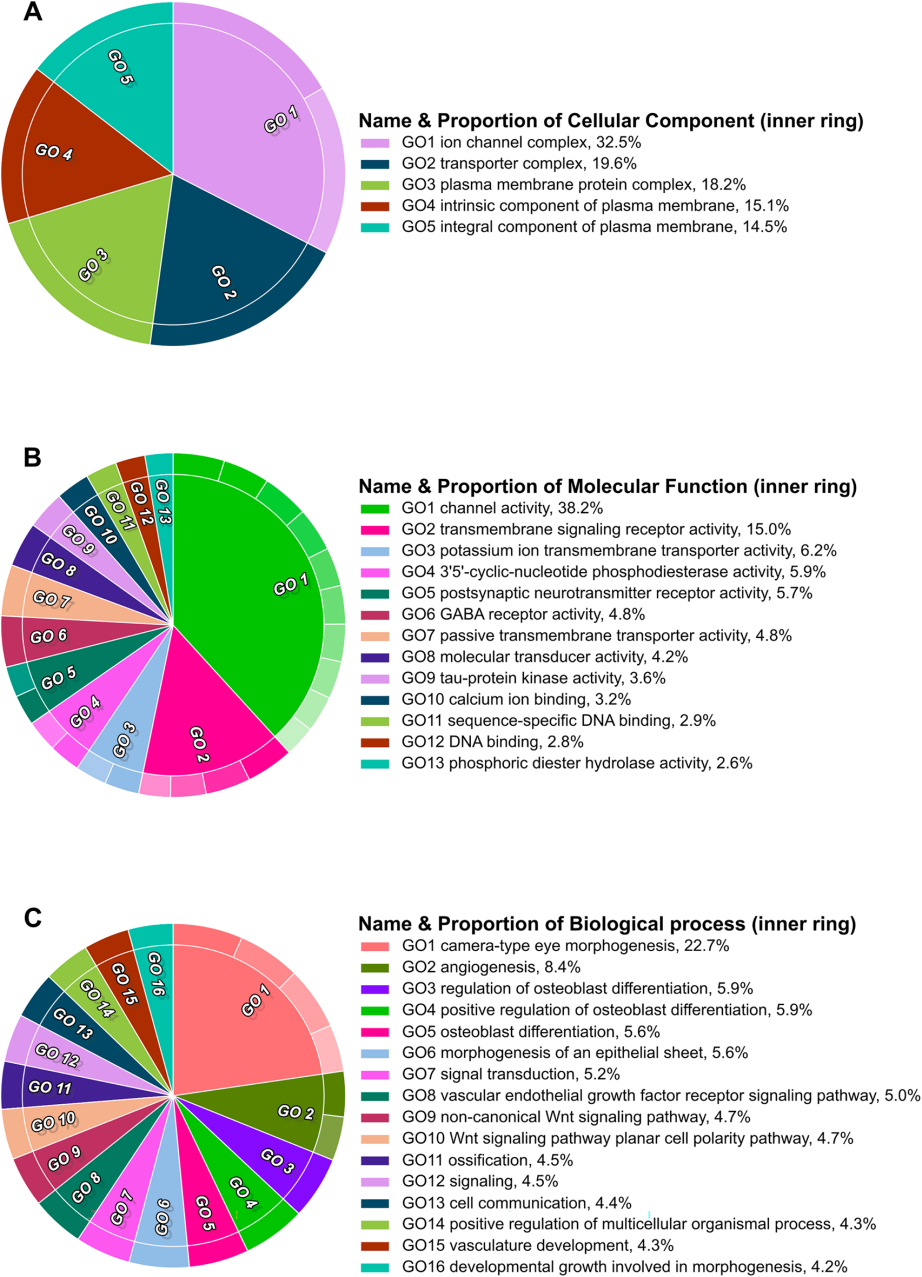
Gene Ontology (GO) terms enriched among the Differentially Expressed (DE) Orthologs between *Coelioxoides waltheriae* and *Tetrapedia diversipes*. Each pie chart represents one of the three main GO categories: (**A**) Cellular Component, (**B**) Molecular Function, and (**C**) Biological Process. Legends next to each chart indicate the representative color, name, and proportion of DE that received an annotation related to the parental GO term. Child terms from the second hierarchical level are represented in the outermost part of the pie chart using shades of the parental representative term color. Parent–child GO term relationships are listed in Supplementary Material 4.

Through the Gene Ontology term enrichment analysis for all categories (BP, CC, and MF), we identified 54 GO terms enriched among the DE orthologs (Supplementary Material 4). The redundant terms of each GO category were summarized in a two-level hierarchical list for visualization (Fig. 2). Top enriched Cellular Component (CC) terms were related to ion channel complex, transporter complex, and plasma membrane protein complex. Consistently, top enriched Molecular Function (MF) terms were related to channel activity, transmembrane signaling receptor activity, and potassium ion transmembrane activity. The orthologs annotated with these CC and MF terms are shown in Table 2.

**Table 2:** Differentially expressed (DE) orthologs between *Coelioxoides waltheriae* and *Tetrapedia diversipes* grouped according to their association to the top enriched GO terms for Cell Component and Molecular Function categories.

DEO IDs	Expression patterns	Homologs (Swiss-Prot organism)	GO hierarchical groups
Calcium channels			
OG0000284	*C. waltheriae* ↓ *T. diversipes* ↑	*CAC* (*Apis mellifera*)	*Cell component* GO1 ion channel complex GO2 transporter complex GO3 plasma membrane protein complex *Molecular function* GO1 channel activity GO7 passive transmembrane transporter activity GO10 calcium ion binding
OG0000168	*C. waltheriae* ↓ *T. diversipes* ↑	*Trp* (*Drosophila melanogaster*)	*Cell Component* GO1 ion channel complex GO2 transporter complex GO3 plasma membrane protein complex GO4 intrinsic component of plasma membrane GO5 integral component of plasma membrane *Molecular Function* GO1 channel activity GO7 passive transmembrane transporter activity
Sodium channels			
OG0003129	*C. waltheriae* ↑ *T. diversipes* ↓	*NaCP60E* (*Drosophila melanogaster*)	*Cell component* GO1 ion channel complex GO2 transporter complex GO3 plasma membrane protein complex GO4 intrinsic component of plasma membrane GO5 integral component of plasma membrane *Molecular function* GO1 channel activity GO7 passive transmembrane transporter activity
OG0002153	*C. waltheriae* ↑ *T. diversipes* ↓	*unc80* (*Drosophila melanogaster*)	*Cell component* GO1 ion channel complex GO2 transporter complex *Molecular Function* GO1 channel activity GO7 passive transmembrane transporter activity
Potassium channels			
OG0004503	*C. waltheriae* ↓ *T. diversipes* ↑	*KCNQ1* (*Felis catus, Xenopus laevis*)	*Cell component* GO1 ion channel complex GO2 transporter complex GO3 plasma membrane protein complex GO4 intrinsic component of plasma membrane GO5 integral component of plasma membrane *Molecular function* GO1 channel activity GO3 potassium ion transmembrane transporter activity GO7 passive transmembrane transporter activity
OG0000260	*C. waltheriae* ↓ *T. diversipes* ↑	*Sh* (*Drosophila melanogaster*)	*Cell component* GO1 ion channel complex GO2 transporter complex GO3 plasma membrane protein complex GO4 intrinsic component of plasma membrane GO5 integral component of plasma membrane *Molecular function* GO1 channel activity GO3 potassium ion transmembrane transporter activity GO7 passive transmembrane transporter activity
OG0004153	*C. waltheriae* ↑ *T. diversipes* ↓	*KCNT1* (*Homo sapiens*)	*Molecular function* GO1 channel activity GO3 potassium ion transmembrane transporter activity GO7 passive transmembrane transporter activity
OG0002734	*C. waltheriae* ↑ *T. diversipes* ↓	*KCNK9* (*Homo sapiens, Rattus norvegicus*)	*Molecular function* GO1 channel activity GO3 potassium ion transmembrane transporter activity GO7 passive transmembrane transporter activity
Chloride channel			
OG0000326	*C. waltheriae* ↓ *T. diversipes* ↑	*Grd* (*Drosophila melanogaster*)	*Molecular function* GO1 channel activity GO2 transmembrane signaling receptor activity GO6 GABA receptor activity GO7 passive transmembrane transporter activity GO8 molecular transducer activity
Ligand-gated ion channels			
OG0000029	*C. waltheriae* ↓ *T. diversipes* ↑	*Grik2* (*Mus musculus*, *Xenopus laevis*)	*Cell component* GO1 ion channel complex GO2 transporter complex GO3 plasma membrane protein complex GO4 intrinsic component of plasma membrane GO5 integral component of plasma membrane *Molecular function* GO1 channel activity GO2 transmembrane signaling receptor activity GO3 potassium ion transmembrane transporter activity GO5 postsynaptic neurotransmitter receptor activity GO7 passive transmembrane transporter activity GO8 molecular transducer activity
OG0000962	*C. waltheriae* ↓ *T. diversipes* ↑	*nAChRβ1* (*Drosophila melanogaster*)	*Cell component* GO1 ion channel complex GO2 transporter complex GO3 plasma membrane protein complex GO4 intrinsic component of plasma membrane GO5 integral component of plasma membrane *Molecular function* GO1 channel activity GO2 transmembrane signaling receptor activity GO5 postsynaptic neurotransmitter receptor activity GO7 passive transmembrane transporter activity GO8 molecular transducer activity
G-protein coupled receptors			
OG0002068	*C. waltheriae* ↓ *T. diversipes* ↑	*GABBR1* (*Homo sapiens*, *Mus musculus*)	*Cell component* GO3 plasma membrane protein complex GO4 intrinsic component of plasma membrane GO5 integral component of plasma membrane *Molecular function* GO2 transmembrane signaling receptor activity GO5 postsynaptic neurotransmitter receptor activity GO6 GABA receptor activity GO8 molecular transducer activity
OG0001556	*C. waltheriae* ↑ *T. diversipes* ↓	*Gabbr2* (*Mus musculus*)	*Cell component* GO3 plasma membrane protein complex GO4 intrinsic component of plasma membrane GO5 integral component of plasma membrane *Molecular function* GO2 transmembrane signaling receptor activity GO6 GABA receptor activity GO8 molecular transducer activity
OG0000063	*C. waltheriae* ↑ *T. diversipes* ↓	*Octβ2R* (*Drosophila melanogaster*)	*Molecular function* GO2 transmembrane signaling receptor activity GO8 molecular transducer activity
OG0001943	*C. waltheriae* ↑ *T. diversipes* ↓	*HTR2C* (Mus musculus, *Bos taurus*)	*Molecular function* GO2 transmembrane signaling receptor activity GO8 molecular transducer activity
OG0000814	*C. waltheriae* ↓ *T. diversipes* ↑	*CG31760* (*Drosophila melanogaster*)	*Molecular function* GO2 transmembrane signaling receptor activity GO8 molecular transducer activity
OG0001490	*C. waltheriae* ↓ *T. diversipes* ↑	*Gr64f./Gr5a* (*Drosophila melanogaster*)	*Molecular function* GO2 transmembrane signaling receptor activity GO8 molecular transducer activity
G protein			
OG0004442	*C. waltheriae* ↓ *T. diversipes* ↑	*G-sα60A* (*Anopheles gambiae*)	*Cell component* GO3 plasma membrane protein complex
Translocases			
OG0001155	*C. waltheriae* ↑ *T. diversipes* ↓	*Atpα* (*Drosophila melanogaster*)	*Cell component* GO2 transporter complex GO3 plasma membrane protein complex GO4 intrinsic component of plasma membrane GO5 integral component of plasma membrane *Molecular function* GO1 channel activity GO3 potassium ion transmembrane transporter activity
OG0001472	*C. waltheriae* ↑ *T. diversipes* ↓	*nrv2/nrv1* (*Drosophila melanogaster*)	*Cell component* GO2 transporter complex GO3 plasma membrane protein complex GO4 intrinsic component of plasma membrane GO5 integral component of plasma membrane
OG0000864	*C. waltheriae* ↓ *T. diversipes* ↑	*UQCRFS1* (*Lagothrix lagotricha*, *Colobus polykomos*)	*Molecular function* GO1 channel activity
DNA-binding protein			
OG0003803	*C. waltheriae* ↑ *T. diversipes* ↓	*Hr38* (*Drosophila melanogaster*)	*Molecular function* GO2 transmembrane signaling receptor activity GO8 molecular transducer activity GO11 sequence-specific DNA binding GO12 DNA binding
Low-density lipoprotein-receptor-related			
OG0004128	*C. waltheriae* ↑ *T. diversipes* ↓	*LRP6* (*Homo sapiens*, *Mus musculus*)	*Cell component* GO3 plasma membrane protein complex *Molecular function* GO2 transmembrane signaling receptor activity GO8 molecular transducer activity
Cell adhesion molecules			
OG0000727	*C. waltheriae* ↑ *T. diversipes* ↓	*NRXN1* (*Homo sapiens*, *Mus musculus*)	*Cell component* GO4 intrinsic component of plasma membrane GO5 integral component of plasma membrane *Molecular function* GO2 transmembrane signaling receptor activity GO8 molecular transducer activity GO10 calcium ion binding
OG0000165	*C. waltheriae* ↑ *T. diversipes* ↓	*Stan* (*Drosophila melanogaster*)	*Molecular function* GO2 transmembrane signaling receptor activity GO8 molecular transducer activity GO10 calcium ion binding
Protein phosphatase			
OG0000836	*C. waltheriae* ↓ *T. diversipes* ↑	*Ptp69D* (*Drosophila melanogaster*)	*Molecular function* GO2 transmembrane signaling receptor activity GO8 molecular transducer activity
Tyrosine-protein kinase			
OG0002698	*C. waltheriae* ↓ *T. diversipes* ↑	*Abl* (*Drosophila melanogaster*)	*Molecular function* GO2 transmembrane signaling receptor activity GO8 molecular transducer activity

The orthologs are represented by the ID of their respective orthogroups. Expression patterns of orthologs comparatively between *C. waltheriae* and *T. diversipes* are indicated by arrows: ↑ (upregulated) and ↓ (downregulated). GO hierarchical groups refer to CirGo terms as shown in Fig. 2.

For the Biological Process (BP) category, the most significant terms in the enrichment analysis were related to camera-type eye morphogenesis, angiogenesis, and regulation of osteoblast differentiation. The main DE orthologs annotated within these terms were identified as homologs of *Fibrillin-2-like*, *Low-density lipoprotein receptor-related protein 6,* and serine/threonine-protein kinases. Moreover, the highest number of DE orthologs were associated with cell communication, signaling, and signal transduction BP terms (Supplementary Material 5).

## Discussion

We employed comparative transcriptomics to investigate molecular distinctions during the reproductive stage of *Coelioxoides waltheriae* (a cleptoparasite) and its host species, *Tetrapedia diversipes*. The present study represents an initial effort to obtain some insights about the molecular mechanisms involved in bee cleptoparasitism by comparing orthologs expression data between a cleptoparasitic species and their host, both inhabiting the same location and at equivalent developmental stages. Considering the constraints of cross-species transcriptomic comparisons, our findings shed light on broad molecular variations between the cleptoparasite and its host, suggesting possible ecological implications.

We overlapped two statistical approaches to perform a cross-species comparison and to reduce the number of false positives in our analyses. The first of these methods was the SCBN, a recently proposed method for count normalization optimized to deal with cross-species comparisons^46^. Secondly, we used a traditional differential expression workflow with the edgeR Bioconductor package^45^. By overlapping the results from these two approaches we consistently retrieved 646 differentially expressed orthologs between the two species, with PiggyBac Transposable Element-derived like (*Pgbd-like*) genes being the most frequent (Fig. 1). Overall, we found many orthologs annotated as *Pgbd-like,* and several of them were found to be highly expressed in *C. waltheriae*. The PiggyBac transposon superfamily is widespread among eukaryotes. The first PiggyBac element was described from a cell culture of *Trichoplusia ni* (Lepidoptera: Noctuidae), the cabbage looper moth^49,50^. Since then, several PiggyBac-like sequences have been described in a variety of organisms. Some of these transposable elements lost transposase activity and are called domesticated^51^. Molecular domestication is a process that occurs due to a transposition inactivating mutation, resulting in the loss of mobility of the transposable element^52^. These insertions may lead to the emergence of new cellular activities, either by altering the coding and/or regulatory regions in which these elements are inserted in the genome or by the evolution of the former TE genes into new genes^52,53^. Thus, these findings lead us to hypothesize whether the domestication of PiggyBac-like transcripts and its regulatory consequences could be one of the molecular mechanisms involved in the evolution and adaptation of *C. waltheriae*, justifying their differential expression between the studied host-parasite species. Indeed, in a recent study, it was observed through comparative genomic analyses that retroviral or transposable elements have undergone a recent or ongoing spread in the genome of a Nomadinae cleptoparasite^54^. This indicates a possible involvement of TEs in the evolution of cleptoparasitism. Future research should further explore this hypothesis, investigating the evolution of transposable and transposable-derived elements in host-parasites genomes and their expression pattern in other cleptoparasitic species of Apidae.

We also identified two MRJP/Yellow-like sequences as highly expressed in *T. diversipes* while none were upregulated in *C. waltheriae*. The MRJP/Yellow gene family encodes multifunctional yellow-like proteins identified in arthropods and in several bacteria^55,56^. Nonetheless, the MRJP-like genes, as part of this family, seem to be restricted to Hymenoptera^56^. Even though these genes have been associated with olfactory learning—particularly regarding *mrjp1* expression in the mushroom body (Kenyon cells)^31,57,58^—and functional studies have shown that MRJPs may have immunoregulatory and antibacterial effects^56^, in bees, MRJPs are mostly associated with larvae feeding, development and with the regulation of phenotypic plasticity and age-polyethism in workers^59^. In honeybees, MRJPs are known to be essential components of the larvae diet, with MRJP1–3 and 5 being the most abundant proteins of larval food^56^. These proteins are synthesized by honeybee nurse workers in the specialized hypopharyngeal glands and offered to the immature offspring through a special food jelly (royal jelly)^60^. In contrast to honeybees, bumblebees do not produce royal jelly. However, their hypopharyngeal glands express one MRJP ortholog^61^. The production of MRJP is not dependent on brood-feeding activity in bumblebees, and protein signals were identified in the abdominal parts of the digestive tract in queens and workers. This suggests that bumblebee MRJP does not have a nutritional function but is instead involved in food digestion and/or modification, consistent with the putatively ancient function of bee hypopharyngeal glands in food digestion^61^. In solitary bees the role of MRJP-like genes is currently poorly understood. One hypothesis for the high expression of MRJPs in *T. diversipes* could be the association of these proteins with larval food. As a solitary bee species, founder females of *T. diversipes* are also responsible for larvae food provisioning and an increased expression of transcripts related to MRJP-like genes could suggest that these proteins may be component of larval food. Yet, the role of MRJPs-like may not be necessarily nutritional/developmental, as they could alternatively play an antibacterial role and/or aid in the processing of bee products (e.g., formation of pollen-pellets and pollen-bread)^62–64^.

Several Biological Process (BP) GO terms enriched among DE orthologs were found to be typical or exclusively related to vertebrate species, such as camera-type eye morphogenesis, angiogenesis, and osteoblast differentiation. It is important to note that molecular data banks, including UniRef, predominantly rely on scientific evidence derived from a limited number of model species^65^. As a result, the nature and extent of GO annotations reflect the aspects investigated in these organisms being only extrapolated to other non-model species under the assumption that putative orthologs are functionally equivalent^66^. However, when dealing with species that are evolutionarily distant, orthologs may have completely different biological functions^67^. Therefore, extrapolating the biological significance of these top enriched BP terms to the invertebrate species investigated in this study is challenging. On the other hand, the enriched Molecular Functions (MF) and Cellular Components (CC) GO terms results were more relatable. The top enriched GO terms for the CC category were related to “ion channel complex”, “transporter complex”, and “plasma membrane protein complex”. Consistently, top enriched MF terms were related to “channel activity”, “transmembrane signaling receptor activity”, and “potassium ion transmembrane activity”. Most of the DE orthologs annotated along with these terms (Table 2) express proteins with a variety of functional roles that are putatively relevant for cleptoparasitic adaptations. In summary, in *C. waltheriae* upregulated orthologs annotated to these terms were related to olfaction, learning, neuronal excitability, synaptic organization, and other neuronal processes. Adaptations in these genetic pathways could be related to the successful exploitation of host resources and the development of specialized behaviors in cleptoparasitic bees. Meanwhile, orthologs highly expressed in *T. diversipes* were involved in chemosensory and sensory perception, particularly gustatory receptor genes involved in sweet taste response, motor, and mitochondrial activity, as well as neurotransmission, genetic mechanisms that could be associated with foraging and nest provisioning performed by *T. diversipes* foundresses. In boxes 1 and 2, we detailed the differentially expressed orthologs associated with the top MF and CC GO terms enriched by addressing their putative functions.

Cleptoparasitic bees are thought to rely on olfactory signals to locate host nests^68,69^. Indeed, morphological differences in antennae sensillae composition have been observed between cleptoparasites and their host species, with cleptoparasites having a greater prevalence of olfactory structures^70^. Previous studies have also shown that chemosensory-related genes (CRGs) are highly expressed in hymenopteran parasitoids^71–74^. Chemosensory cues, along with other external stimuli, are processed in sensory systems, triggering the formation of both long-term and short-term memories^75^. These adaptations may contribute to the localization and memorization of host nest aggregations^76^. In addition to the sensory-related genes described in box 1, we identified other potential chemosensory-related genes (CRGs) upregulated in the cleptoparasite, including Ionotropic Receptors (IRs), a gene family involved in olfaction, gustation, and other sensory perceptions^77,78^. Unlike many other chemosensory gene families, IRs exhibit a remarkable degree of conservation across species^79^. The *IR25a* homolog was found highly expressed in *C. waltheriae*. This gene, along with *IR8a*, functions as a co-receptor (IRco) for the formation of functional IR complexes in conjunction with a ligand-binding receptor protein (IR X)^80^. Furthermore, we detected an upregulated putative odorant receptor (OR) homologous to the *Polistes dominula Or85c-like* sequence in *C. waltheriae*. Although this specific OR's role in odour perception remains unknown, computational analysis in *P. dominula* suggests its involvement in parasitoid sensory perception. It is important to note that our current approach may have missed lineage-specific CRGs, and further investigation into these lineage-specific CRGs in cleptoparasitic lineages is crucial to gain insights into the underlying processes of parasitism in bees.

*C. waltheriae* and *T. diversipes* lineages, both belonging to the Apidae family, diverged over 77 million years ago^11^. Thus, compared to several cleptoparasites, that prefer hosts from other bee families (e.g., cleptoparasites of the Nomadinae clade)^10^, the phylogenetic proximity between *C. waltheriae* and *T. diversipes* could minimize the “phylogenetic noise” in transcriptomic comparisons. Still, cross-species transcriptomic comparisons present challenges, and certain caveats should be considered in the light of the present results. First, our analytical strategy focused on conserved orthologs, thus neglecting non-orthologous or rapidly evolving genes that may play a crucial role in cleptoparasitism evolution. Additionally, due to the divergent life history trajectories of these two species, the differentially expressed orthologs identified could be associated with non-behavioral or random species-specific adaptations and not directly related to cleptoparasitic adaptations. Thus, we argue that applying the methodology used in this study to other bee host-parasite species pairs should allow the differentiation of species-specific adaptations from shared molecular mechanisms. Lastly, we conducted a broad-scale transcriptome analysis, meaning that the RNA-Seq data used were obtained from whole-body extractions. As an exploratory analysis, this approach allowed some initial broad insights, but it is limited in capturing genes with tissue-specific expression or complex expression patterns across the body^81^. We also used sample replicates containing a variable number of pooled individuals for *C. waltheriae*, to account for this sampling strategy we normalized and treated the gene expression counts for batch effects across replicates, and this treatment might have reduced even further our power to detect genes with subtle expression differences. Further research should therefore delve into the obtained results by investigating the expression of these genes in a tissue-specific context, in order to elucidate their functions more accurately. Finally, we analyzed a specific life stage of each species by comparing females performing reproductive activities. We considered that this stage comprises some of the most distinguishing behavioural differences between the host and cleptoparasite species, yet comparative analyses of multiple life stages could provide a more detailed profile of cleptoparasitism across developmental stages.

## Box 1: Orthologs upregulated in *C. waltheriae* and associated to top enriched GO terms

In *C. waltheriae*, we found upregulated orthologs that may have roles in the olfactory and learning pathways, potentially representing adaptations to cleptoparasitic behavior^68,69^.

Among the upregulated sequences in *C. waltheriae*, we discovered homologs to the invertebrate *Octβ2R* and vertebrate *HTR2C*, receptors for biogenic amines octopamine (OA) and serotonin (5-HT), respectively. Biogenic amines play important physiological roles in organisms, modulating neuronal, metabolic, and physiological processes^82^. In hymenopteran species, OA has been linked to locomotor activity, sensory (gustatory, olfactory, and visual) sensitivity, aggressive behavior, and (associative and non-associative) learning^76,83,84^. *Octβ2R* has been associated with rewarding reinforcement signaling in *Drosophila*, indicating its potential role in behavioral responses^85^. In honeybees, octopaminergic signaling is implicated in appetitive learning^86^. Overexpression of octopamine receptors in honeybees has also been associated with oxidative stress, neuroinflammation, and olfactory dysfunction^87^. Similarly, 5-HT acts as a neurotransmitter, neuromodulator, and hormone in insects^88^, regulating various behaviors such as aggression, mating, feeding, locomotion, and olfaction^89–95^. Invertebrate 5-HT receptors are found in the central and peripheral nervous systems (CNS and PNS, respectively), mediating both excitatory and inhibitory actions^96,97^. The signaling of 5-HT receptors in *Drosophila* is linked to aggression, sleep, circadian behavior, feeding, mating, learning, and memory^98–103^.

We also observed upregulation of the subunit of a neurotransmitter receptor in *C. waltheriae*, a homolog of the mammalian *Gabbr2* (Gamma-aminobutyric acid type B receptor subunit 2). Meanwhile the *Gabbr1* (Gamma-aminobutyric acid type B receptor subunit 1) was upregulated in *T. diversipes*. Gamma-aminobutyric acid (GABA) is the major neurotransmitter for inhibitory synaptic transmissions in the CNS of both vertebrates and invertebrates^104,105^. GABA signaling influences insect behaviors such as learning and memory, locomotor activity, and odor processing^106–110^. GABA_B_ receptors, coupled with ionotropic GABA_A_ receptors, mediate the action of GABA^111^. These receptors regulate complex behaviors and nervous system functions by inhibiting GABA release and reducing the release of other neurotransmitters^112^. For instance, in *Drosophila* and *Heliothis virescens*, GABA_B_ receptors are involved in the olfactory pathway^89,113^. In *Apis mellifera*, GABA_B_ is associated with locomotor behavior^106^.

In *C. waltheriae*, we also observed the upregulation of *NaCP60E*, a voltage-gated sodium channel. This gene has been mainly associated with olfactory function, but it is also expressed in other tissues^114,115^ and in invertebrates without olfaction^116^. In *Drosophila*, decreased expression of *NaCP60E* has been shown to decrease the olfactory response to benzaldehyde^117^. Also, a dense concentration of NaCP60E proteins in neuron axons of chemosensory organs suggests that this gene is involved in the olfactory system^118^. Thus, the upregulation of *NaCP60E* suggests its involvement in olfaction-related processes in the cleptoparasitic bee.

*C. waltheriae* also showed increased expression of potassium channel orthologs, specifically homologs of human *KCNT1* and *KCNK9* genes. *KCNT1* encodes a sodium-gated potassium channel that is activated by neuromodulators^119^. Therefore, it is suggested that it regulates neuronal excitability and may play a role in several behaviors^120^. *KCNK9* encodes a two-pore domain potassium channel that is involved in resting membrane potentials^121^. It can also be a target for modulatory molecules, such as volatile anesthetics and neurotransmitters^122^. Additionally, in *Drosophila*, *KCNK9* orthologs (*Task6* and *Task7*) are preferentially expressed in the antennal lobes and may play a role in olfactory memory formation^123^.

Furthermore, we identified in *C. waltheriae* the upregulation of genes associated with neuronal excitability, including circadian rhythm, memory formation, and development. These include *Unc80*, *Hr38*, and *Lrp6*. Unc80 is a regulatory component of the sodium leak channel NALCN complex^124^, which play a role in modulating resting membrane potential, neuronal excitability, firing rates, and pacemaker activity^125,126^. In *Drosophila*, for instance, *Unc80* is necessary for circadian rhythmicity^124^. It is thought that Unc80 may also be involved in bee learning and memory^31^. The gene *Hr38, a nuclear receptor*, is involved in the transcriptional control of the dopamine synthesis pathway^127^, the cuticle gene expression^128^, and the ecdysteroid signaling pathways^129^. In addition, it has been suggested that *Hr38* may play an important role in high neuronal functions such as memory formation, courtship behavior, and circadian rhythm^130–132^. *Lrp6* belongs to the low-density lipoprotein receptor family of cell surface receptors and is an essential component of the Wnt signaling pathway, which controls several biological processes throughout the development and adult life of metazoans^133^. Expression of the *Drosophila* ortholog of *Lrp6* (*arr*) has been associated with CNS morphogenesis and organization^134^, as well as long-term memory formation^135^.

We also found upregulated orthologs of cell adhesion molecules (CAMs) genes, including *NRXN1* and *Fmi/Stan*. In *Drosophila*, *NRXN1* gene is required for proper organization and growth at neuromuscular junctions^136^, and it also regulates sleep and synaptic plasticity^137^. In honeybees, the expression of *NRXN1* is increased under sensory stimulation, suggesting a link between sensory processing and associative learning^138^. *Drosophila Fmi*/*Stan* encodes a cadherin that regulates planar cell polarity^139^, axon guidance in photoreceptor neurons^140^, dendrite morphogenesis of sensory neurons^141^, and neuronal morphogenesis of the mushroom body^142^. The upregulation of CAMs genes can be involved with their role in synaptic organization and plasticity, which may be relevant to the cleptoparasitic lifestyle of *C. waltheriae*.

Lastly, we found upregulated orthologs of *ATPα* and nrv2/nrv1 genes in *C. waltheriae*, which encode components of the Na + /K + ATPase^143,144^. Na + /K + ATPase is important for maintaining the balance of sodium and potassium ions across the cell membrane^145^. Defects in the sodium–potassium pump can lead to neuronal dysfunctions, such as circadian rhythm disturbances, locomotor problems, and auditory mechanosensation^146–148^.

## Box 2: Orthologs upregulated in *T. diversipes* and associated to top enriched GO terms

*T. diversipes* exhibited upregulated sequences related to chemosensory perception, particularly in the context of gustatory receptor (GR) genes. One such sequence was identified in *T. diversipes* as the *Gr5a* gene while in *C. waltheriae*, the corresponding sequence was identified as the *Gr64f.* gene. Despite the difference in annotation, both genes belong to the same conserved subfamily that is involved in the sweet taste response observed in *Drosophila*^149^. Nevertheless, they are located on different chromosomes in *Drosophila* (*Gr5a*: X chromosome; *Gr64f.*: third chromosome)^150^. These receptors are considered the primary basis for sugar reception in *Drosophila*, and their co-expression in gustatory receptor neurons is necessary for the response to certain sugars^149,151^. Since bees primarily rely on flower nectar as an energy source, sugar detection is crucial for their survival^152^. Thus, a higher expression of these receptors in *T. diversipes* may be attributed to their intense foraging activity while provisioning food in their nests, an activity not performed by the parasite. Another upregulated sequence in *T. diversipes* was also related to GR, the transcript homologous to the *G-sα60A* gene of *Anopheles gambiae*. Studies on female antennae of *A. gambiae* have suggested that *G-sα60A* isoforms participate in olfactory signal transduction^153^. It has also been proposed that *Gsα* in *Drosophila* is responsible for Gr5a-mediated sweet taste perception^154^.

Apart from GR genes, several other upregulated genes in *T. diversipes* were associated with sensory perception but were notably involved in distinct pathways when compared to those upregulated in *C. waltheriae*. For example, the *Drosophila Sh* (*Shaker*) gene encodes a voltage-gated potassium channel (Kv)^155^ that is involved in shaping and firing the action potential^156^. It is also expressed in the retina and different regions of the CNS, including the mushroom body neuropil^157,158^. Furthermore, evidence suggests that *Sh* regulation affects olfactory learning and memory^159^. Another putative Kv upregulated in *T. diversipes* was a homolog to the vertebrate *KCNQ1* gene, which regulates essential physiological processes all over the body^160^. In *Drosophila*, *KCNQ1* ortholog (*dKCNQ*) is expressed in the brain cortical neurons, cardia, and in the nurse cells and oocytes in the ovary^161,162^. Apart from its role in the fly's normal heartbeat^162^ and early embryonic development^161^, *dKCNQ* has been implicated in age-dependent memory impairment and is required in α/β Mushroom Body Neurons for setting short-term memories^163^.

Another upregulated sequence in *T. diversipes*, homologous to the *Drosophila trp* (Transient Receptor Potential) gene, may also be related to sensory perception. TRP proteins have been associated with phototransduction and olfactory response to CO_2_ in *Drosophila*^164,165^.

An upregulated homolog of the *Drosophila abl* (Abelson) gene, which encodes a non-receptor tyrosine kinase, was also identified upregulated in *T. diversipes*. The *Drosophila abl* gene is expressed in the axons of the CNS and plays a very important role in establishing axonal connections during CNS development^166^. Also, it has been suggested that Abl-mediated phosphorylation is an important mechanism for the fly visual development^167^.

Motor activity-related genes also exhibited higher expression levels in *T. diversipes*. One such gene is the *cacophony* (*Cac*), which encodes a voltage-gated calcium channel that plays a role in regulating neurotransmitter release at neuromuscular synapses in *Drosophila*^168^. Similarly, a homolog of the *Drosophila Ptp69D* gene, which encodes a receptor of tyrosine phosphatase that is associated with mechanosensory neuron development^169^, was also upregulated gene in *T. diversipes*. *Ptp69D* might influence some elements of motor function in adult *T. diversipes* females.

Additionally, in *T. diversipes,* there was a higher expression of a homolog to the *UQCRFS1* gene. This gene encodes an iron-sulfur protein (Rieske Fe-S protein—RISP)^170^ and is involved in electron transfer in bc1 complex^171^. Furthermore, *T. diversipes* displayed higher expression of homologs of other genes associated to electron transport activity, including genes with mitochondrial activity, indicating an increased energy demand during the founder life stage. These upregulated genes included, *ETFA*, *MRPS18C*, *mdh2*, *Cox6al*, and *Ndufs2*. The *ETFA* gene encodes an electron transfer flavoprotein subunit alpha, which is involved in mitochondrial fatty acid beta-oxidation and amino acid catabolism^172^. *MRPS18C* encodes a mitochondrial ribosomal protein that plays a role in protein synthesis within mitochondria^173^. The *mdh2* gene encodes a malate dehydrogenase, an enzyme involved in the citric acid cycle^174^. *Cox6al* encodes a subunit of cytochrome c oxidase^175^, and *Ndufs2* encodes a core subunit of NADH dehydrogenase (Complex I)^176^, another two of the critical enzymes in the electron transport chain.

Finally, several upregulated genes in *T. diversipes* were homologous to neurotransmitter receptors, including *nAChRβ1*, *Grik2*, and *Grd*. The *nAChRβ1* gene encodes a subunit of the nicotinic acetylcholine receptor (nAChR), which is the primary excitatory neurotransmitter in insects. It is involved in olfactory learning and memory formation, as well as modulation of aggressive behavior^177,178^. *Grik2* encodes the vertebrate glutamate receptor ionotropic kainate 2, which contribute to rapid synaptic transmission^179^. However, the role of kainate receptors in CNS glutamatergic circuits of insects is not well understood^180^. The *Grd* gene encodes a *Drosophila* chloride-channel homolog of the mammalian GABA receptor delta subunit that may respond to different neurotransmitters^181^. This gene has been linked to mediating the glycine response^181^ and forming functional ionotropic GABA receptors^182^. Additionally, an upregulated homolog of the *Drosophila CG31760* gene was found in *T. diversipes*. *CG31760* is a probable G-protein coupled receptor (GPCR), whose expression was highest in the adult fly brain however its exact function is unknown^183^.

## Conclusion

In conclusion, our study provides global insights into the molecular mechanisms of cleptoparasitism in bees. We observed differential gene expression between the cleptoparasite *C. waltheriae* and its host *T. diversipes* particularly involving genes related to signal transduction, sensory perception, learning, and memory formation. These findings suggest the importance of sensory adaptations and learning in host-parasite interactions. We also identified a higher abundance of transcripts derived from transposable elements in *C. waltheriae* transcriptome, indicating these could be involved in gene neofunctionalization for parasite-specific adaptations. Additionally, the host species exhibited highly expressed genes from the Major Royal Jelly Proteins family. In bees, these genes are associated with various functions, including nutritional, immunity, and developmental regulation. We hypothesize that the differential expression of these genes could be related to nest provisioning in *T. diversipes*, a task not performed by the parasite. Further investigation is required to fully understand the role of all these molecular mechanisms in cleptoparasitism and their potential tissue-specific functions. Moreover, we propose that the methodology employed in the present study should be extended to other bee host-parasite species pairs, allowing for better differentiation of species-specific adaptations and shared molecular mechanisms underlying cleptoparasitism and its convergent traits in bees.

## Material and methods

### Sample collection and RNA sequencing

*T. diversipes* samples were collected and sequenced in a previous study^33^. Briefly, females were collected during their reproductive period (November to December of 2012, here called G1, and March to July of 2013, here called G2), at Universidade de São Paulo (23° 33′ 53.2″ S 46° 43′ 51.7″ W), while provisioning their nests. G1 and G2 represent the foundresses of different reproductive generations. Individuals were always sampled between 10:00 A.M. and 12:30 P.M., immediately frozen in liquid nitrogen and stored at − 80 °C. RNA extractions were performed individually using the whole body. Each sample replicated contained pooled RNA from three individuals. Here we included in the analyses RNA-Seq data from five replicates: three from G1 and two from G2. For more details see^33^.

Adult females of *C. waltheriae* were collected at the same location as *T. diversipes* samples while hovering at the entry of *T. diversipes* trap nesting spots. This behaviour is typical of *C. waltheriae* females looking for locations to lay their eggs^21^. Two individuals were collected in March 2013 (along with *T. diversipes* samples), and six more were collected later between November 2015 and March 2016. These individuals were also sampled between 10:00 A.M. and 12:30 P.M., immediately frozen in liquid nitrogen, and stored at − 80 °C. Total RNA extractions were performed for each individual separately using the RNeasy® Mini Kit (Qiagen, Austin, Texas, USA), following the manufacturer’s instructions. RNA was extracted from the whole body of the samples to enable a broad-scale transcriptomic analysis. Quantification and quality assessment of RNA was performed initially using NanoDrop™ Lite (Thermo Fisher Scientific, Wilmington, Delaware, USA) and later with the Agilent 2100 Bioanalyzer system (Agilent Technologies, Palo Alto, California, USA) before the library preparations. *C. waltheriae* samples were divided into five replicates: two containing only one individual in each (samples collected in 2013); and three containing the pooled RNA of two individuals (collected between 2015 and 2016). Library preparation and sequencing of the first two replicates (2013 samples) were performed by Macrogen (Macrogen Inc., Seoul, South Korea), along with *T. diversipes* samples from^33^, using the Illumina® HiSeq 2000 platform. The other three replicates of *C. waltheriae* (sampled from 2015–2016) were sequenced at LaCTAD (Unicamp, Campinas, Brazil), also using an Illumina® HiSeq 2000 sequencer.

*C. waltheriae* RNA sequencing resulted in 285,806,598 raw reads. After cleaning, read number decreased to 169,961,662. For *T. diversipes*, 343,059,264 raw reads were used, resulting in 190,339,784 cleaned reads after trimming.

### Transcripts assembly

Reads quality assessment was performed using the FastQC 0.11.5^184^. Trimmomatic 0.38^185^ was used to quality trimming the raw reads (options: SLIDINGWINDOW:4:30 TRAILING:3 MINLEN:80 AVGQUAL:30 HEADCROP:14). Cleaned reads were then digitally normalized (20 × coverage) using a script (*insilico read normalization*) implemented within the Trinity toolbox^186,187^. Transcriptome assembly was performed differently for each species. *C. waltheriae* transcriptome was assembled following Trinity 2.10.0^186,187^ de novo assembly protocol with default parameters. For *T. diversipes*, a draft genome was available (Santos et al., unpublished), so two different approaches were used: (1) a genome-guided transcriptome assembly, using STAR 2.7.3^188^ and Cufflinks 2.2.1^189^; and (2) a reference guided de novo assembly with Trinity, using the bam file generated by the STAR software. The transcripts resulting from these two assemblies were clustered into SuperTranscripts^190^ by using CD-Hit^191^, Corset^192^, and Lace^190^. This clustering approach was also applied to the de novo assembly of *C. waltheriae*, to make the datasets more comparable between the two species and to reduce transcript redundancy in *C. waltheriae*. Downstream differential expression analyses were performed using the SuperTranscripts non-redundant datasets. Quality parameters of the transcriptomes were analyzed with Transrate 1.0.3^38^ and BUSCO 5.2.2^193^ (using hymenoptera_odb10 database).

### Annotation and expression analysis of orthologs

Non-redundant transcripts were annotated with Annocript 2.0.1^194^ pipeline using the UniProt Reference Clusters (UniRef90)^195^ and UniProtKB/Swiss-Prot^196^ databases from February 2021. Transcripts with significant blast hits (*e*-value < 1e−5) against possible contaminants (i.e., Acari, Alveolata, Archaea, Bacteria, Fungi, Rhizaria, Rhodophyta, Viridiplantae, and Viruses) in the UniRef90 database were removed from the final data set using custom scripts (https://github.com/PauloCseri/Annotation.git).

To identify orthologs between the two species the amino acid sequences of the resulting ORFs predicted in the Annocript pipeline were used as input to Orthofinder 2.1.1^40,41^. Then, we selected the best matching transcripts per species in each orthogroup to get one-to-one ortholog data. This selection was performed by filtering the transcripts of the highest bitscore value (better alignment) between sequences of the two species in the same orthogroup. These alignments were performed using the blastp algorithm^42,43^ with default parameters. Differential expression (DE) analyses were then performed using only the orthologs identified in both species.

To estimate gene expression levels, we used Bowtie 2.3.5.1^197^ to align the cleaned reads to their respective ortholog transcripts set, i.e., *C. waltheriae* sample reads were aligned to *C. waltheriae* ortholog transcripts and *T. diversipes* reads to its corresponding set of ortholog transcripts. Then, we used RSEM 1.3.3^198^ for read counting. The significant DE orthologs between the two species were identified by combining the following approaches: (1) using the edgeR 3.34.0^45^ with TMM normalized counts^44^; and (2) using NOISeq 2.36.0^47,48^ with the normalization factor calculated through the SCBN method to normalize read counts^46^. This normalization strategy is optimized for cross-species DE analyses^46^ and is implemented in the Bioconductor package SCBN 1.10.0. Only significant DE orthologs (|Log2FC|≥ 2, adjusted *p-value* < 0.05) commonly identified as so in these two analyses were selected for the resulting set of DEs. It is worth mentioning that as the samples were not sequenced in the same batch (different years), we additionally adjusted these counts for batch effects using the ComBat-seq method^199^ available on the Bioconductor package sva 3.38.0^200^. In detail, for the batch parameter, we used the different sequencing times as factors (0: initial sequencing of *C. waltheriae* and *T. diversipes* samples; 1: later sequencing of *C. waltheriae* samples only) and for the *group* parameter (biological condition), we used the respective species (0: *C. waltheriae*; 1: *T. diversipes*).

### GO functional analysis

Assuming that sequences from the same orthogroup descend from a single gene in the last common ancestor^40,41^ and hence they likely have similar functions, the ensemble of functional GO annotations from all SuperTranscripts belonging to the same orthogroup were used for functional analyses. The Bioconductor package PloGO2 1.4.0^201^ was used to plot and visualize the GO annotations of DE orthologs. To test whether any GO term was enriched among the DE orthologs in comparison to all other orthologs identified we used the Bioconductor package TopGO 2.20.0^202^. Redundant GO enriched terms were summarized in a two-level hierarchical GO set using the REVIGO web server^203^ for simplification, and these hierarchical sets were represented in charts generated by the CirGO 2.0 software^204^.

### Supplementary Information

Supplementary Information 1.

Supplementary Information 2.

Supplementary Information 3.

Supplementary Information 4.

Supplementary Information 5.

## Data Availability

The raw sequence reads have been deposited in the NCBI Sequence Read Archive (SRA) under the following accession numbers: SRR24187037, SRR24187038, SRR24187039, SRR24187040, SRR24187041, SRR24187042, SRR24187043, SRR24187044, SRR24187045, SRR24187046. The corresponding BioProject accession number is PRJNA955762. The transcriptome assemblies, as well as the sets of SuperTranscripts, are available on FigShare (10.6084/m9.figshare.23264771.v1).
