# Supplementary material for: Decoding bee cleptoparasitism through comparative transcriptomics of Coelioxoides waltheriae and its host Tetrapedia diversipes
Source: Sci Rep. 2024 May 29;14:12361. doi: 10.1038/s41598-024-56261-5 (PMC11137135; doi:10.1038/s41598-024-56261-5)
Supplement: Supplementary file 2 — Supplementary Information 2. [file 41598_2024_56261_MOESM2_ESM.pdf]

Decoding bee cleptoparasitism through comparative transcriptomics of *Coelioxoides waltheriae* and its host *Tetrapedia diversipes*

Paulo Cseri Ricardo[1*], Maria Cristina Arias[1] and Natalia de Souza Araujo[2]

[1] *Departamento de Genética e Biologia Evolutiva – Instituto de Biociências, Universidade de São Paulo, São Paulo, Brazil.*
[2] *Unit of Evolutionary Biology & Ecology – Université libre de Bruxelles, Brussels, Belgium.*
*Corresponding author: P. C. Ricardo - cseri.bio@gmail.com

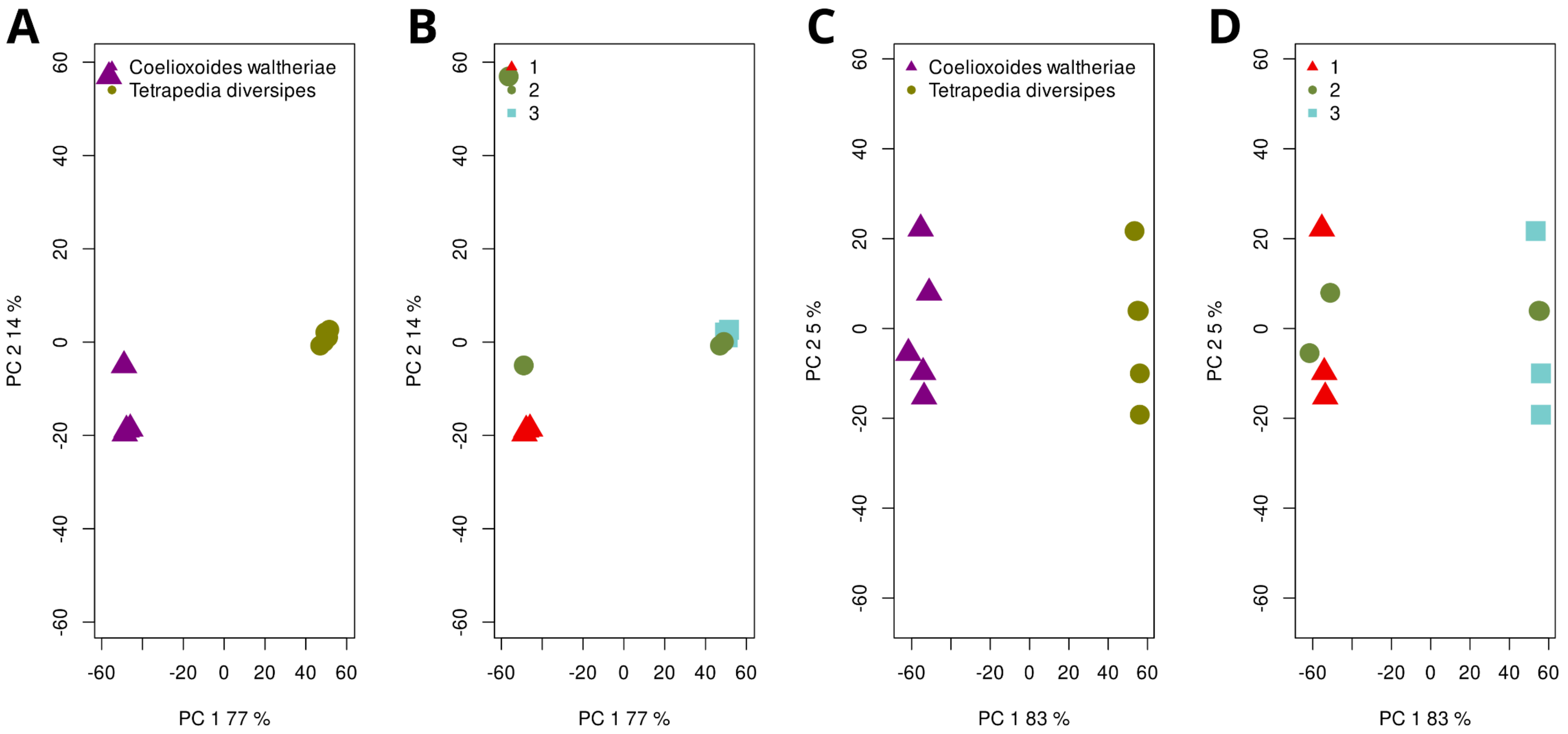

Figure 1. Principal component analysis (PCA) plots showing samples of *Coelioxoides waltheriae* (purple triangles) and *Tetrapedia diversipes* (olive circles) based on gene expression profile, before (A) and after (C) batch correction on raw data. The samples from each batch (batch 1: red triangles; 2: green circles; 3: blue squares) are represented in the same way, before (B) and after (D) correction. The X- and Y-axes represent the first and second principal components (PC1 and PC2) and the associated percentage of variation.
