# Supplementary material for: Decoding bee cleptoparasitism through comparative transcriptomics of Coelioxoides waltheriae and its host Tetrapedia diversipes
Source: Sci Rep. 2024 May 29;14:12361. doi: 10.1038/s41598-024-56261-5 (PMC11137135; doi:10.1038/s41598-024-56261-5)
Supplement: Supplementary file 5 — Supplementary Information 5. [file 41598_2024_56261_MOESM5_ESM.pdf]

Decoding bee cleptoparasitism through comparative transcriptomics of *Coelioxoides waltheriae* and its host *Tetrapedia diversipes*

Paulo Cseri Ricardo[1*], Maria Cristina Arias[1] and Natalia de Souza Araujo[2]

[1] *Departamento de Genética e Biologia Evolutiva – Instituto de Biociências, Universidade de São Paulo, São Paulo, Brazil.*
[2] *Unit of Evolutionary Biology & Ecology – Université libre de Bruxelles, Brussels, Belgium.*
*Corresponding author: P. C. Ricardo - cseri.bio@gmail.com

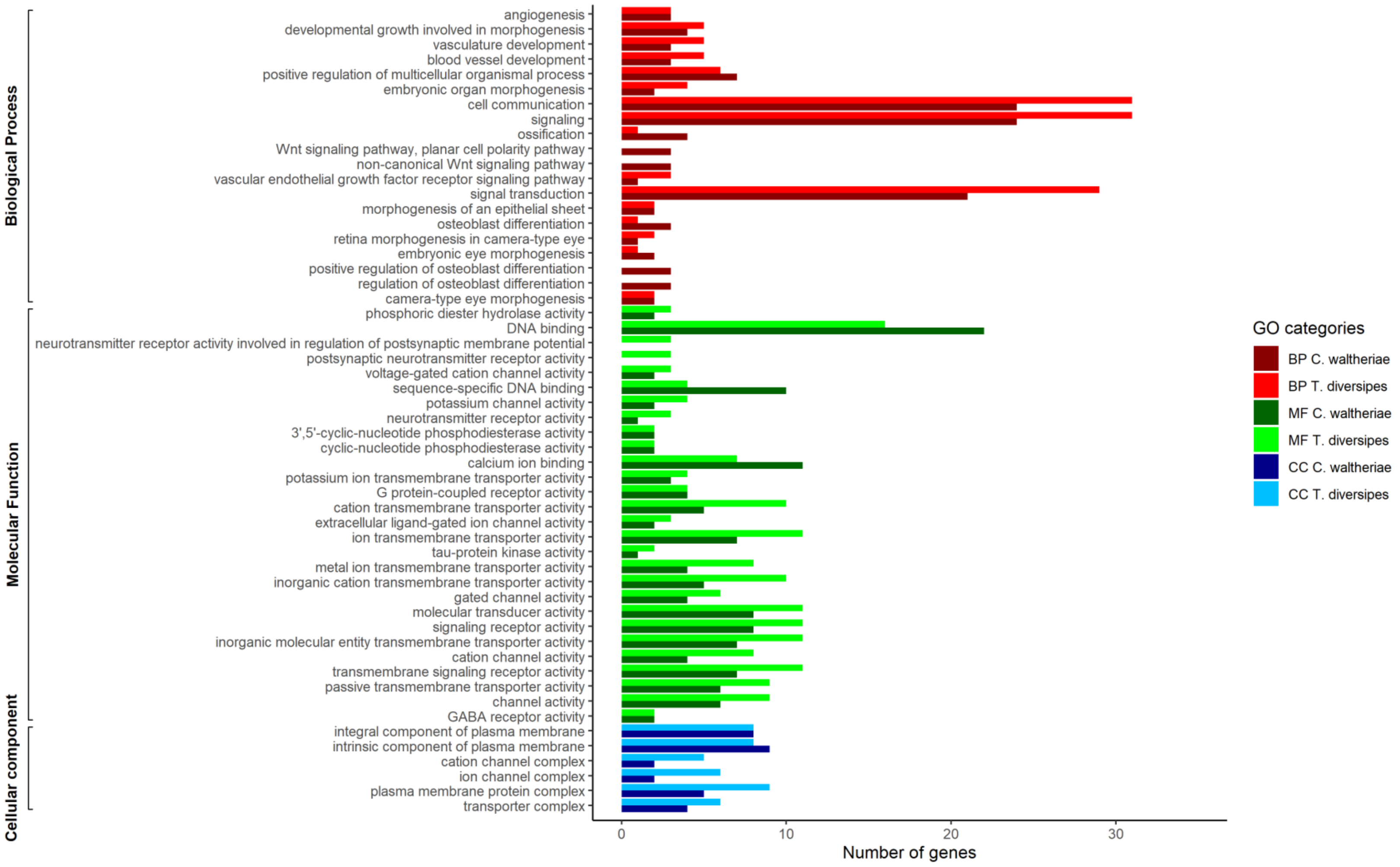

Figure 2. Gene ontology (GO) functional classification of *Coelioxoides waltheriae* (darker) and *Tetrapedia diversipes* (lighter) differently expressed orthologs. The GO functional annotations were classified into three main categories: Cellular Component (blue), Molecular Function (green) and Biological Process (red).
